# Influence of dextran-70 on systemic inflammatory response and myocardial ischaemia-reperfusion following cardiac operations

**DOI:** 10.1186/cc6095

**Published:** 2007-08-14

**Authors:** Károly Gombocz, Ágnes Beledi, Nasri Alotti, Gábor Kecskés, Valéria Gábor, Lajos Bogár, Tamás Kőszegi, János Garai

**Affiliations:** 1 Zala County Hospital, Department of Cardiac Surgery, University of Pécs, Zalaegerszeg, Hungary; 2 Zala County Hospital, Department of Pathology, University of Pécs, Zalaegerszeg, Hungary; 3 Department of Anesthesia and Intensive Care, University of Pécs, Pécs, Hungary; 4 Institute of Laboratory Medicine, University of Pécs, Pécs, Hungary; 5 Department of Pathophysiology and Gerontology, University of Pécs, Pécs, Hungary

## Abstract

**Introduction:**

Experimental studies have demonstrated that dextran-70 reduces the leukocyte–endothelium interaction, but clinical evidence is still lacking. Our objective was to justify the anti-inflammatory effect of dextran-70 following cardiac operations.

**Methods:**

Forty patients undergoing coronary bypass surgery (*n *= 32) or aortic valve replacement (*n *= 8) were enrolled in this prospective, randomized, double-blind study. Two groups were formed. In group A (*n *= 20), dextran-70 infusion was administered at a dose of 7.5 ml/kg before the initiation of cardiopulmonary bypass and at a dose of 12.5 ml/kg after the cessation of cardiopulmonary bypass. Group B served as a control with identical amounts of gelatin infusion (*n *= 20). The plasma concentration of procalcitonin, C-reactive protein, IL 6, IL 6r, IL 8, IL 10, soluble endothelial leukocyte adhesion molecule-1, soluble intercellular adhesion molecule-1, cardiac troponin-I and various haemodynamic parameters were measured in the perioperative period. Multivariate methods were used for statistical analysis.

**Results:**

In group A, lower peak (median) plasma levels of procalcitonin (0.2 versus 1.4, *p *< 0.001), IL 8 (5.6 versus 94.8, *p *< 0.001), IL 10 (47.2 versus 209.7, *p *= 0.001), endothelial leukocyte adhesion molecule-1 (88.5 versus 130.6, *p *= 0.033), intercellular adhesion molecule-1 (806.7 versus 1,375.7, *P *= 0.001) and troponin-I (0.22 versus 0.66, *p *= 0.018) were found. There was no significant difference in IL 6, IL-6r and C-reactive protein values between groups. Higher figures of the cardiac index (*p *= 0.010) along with reduced systemic vascular resistance (*p *= 0.005) were noted in group A.

**Conclusion:**

Our investigation demonstrated that the use of dextran-70 reduces the systemic inflammatory response and cardiac troponin-I release following cardiac operation.

**Trial registration number:**

ISRCTN38289094.

## Introduction

Cardiac surgery on cardiopulmonary bypass (CPB) results in a complex immune response characterized by the activation of all inflammatory pathways and strongly related to increased postoperative morbidity and mortality. The immune activation due to systemic inflammatory response syndrome exposes the patient to postoperative wound healing complications and to the development of infections [1,2].

Increased levels of the proinflammatory cytokines IL-6 and IL-8 play a major role in the pathogenesis of ischaemia-reperfusion injury [3] and multiple organ dysfunction syndrome [4]. IL-8 is a crucial chemokine, which attracts and activates polymorphonuclear leukocytes (PMNs) as well as T lymphocytes, and controls their migration. Tissue penetration, free radical production, and granulocyte elastase synthesis and release are also increased in PMNs [4]. Serum procalcitonin is a sensitive marker for the early detection of systemic inflammatory response syndrome [5]. Procalcitonin levels above 2 ng/ml are predictive for postoperative complications after cardiac operations [6]. Owing to inflammation, soluble adhesion molecules are shed into the circulation and their concentration correlates with the magnitude of endothelial activation and injury [1,7].

Several investigations have demonstrated that artificial colloids modulate the inflammatory response. Animal experiments have confirmed that dextran decreases the endothelial adhesion of PMNs in the postischaemic phase independently of the haemodilution [8]. Among trauma patients, dextran administration counteracts monocyte dysfunction and the related imbalance between coagulation and fibrinolysis [9]. Hypertonic saline dextran suppressed myocardial TNF-α, IL-1β and IL-6 secretion after an initial burn injury in an animal study, improving ventricular performance after subsequent septic challenge [10]. Experimental and clinical studies have justified the beneficial effects of dextran in the prevention of acute respiratory distress syndrome following trauma and sepsis [11], radiation injury [12], pancreatitis [13] and lower limb reperfusion injury [14].

It has been reported that hydroxyethyl starches (HES) reduce capillary leakage [15], leading to the concept of 'plugging the leaks' in various diseases, including sepsis and burns [16]. In a polymicrobial sepsis model, HES inhibited the inflammatory cytokine response, neutrophil infiltration and expression of intercellular adhesion molecule-1 (ICAM-1) mRNA [17]. Other anti-inflammatory manifestations of low-molecular-weight HES include impaired neutrophil respiratory burst and reduced neutrophil chemotaxis [18]. Administration of HES was associated with reduced markers of inflammation and endothelial activation in sepsis [19] and in patients undergoing major abdominal surgery [20].

Without any evidence of modulating the inflammatory response, gelatin infusion has been considered pharmacologically inert [21]. This assumption was confirmed in an experimentally induced acute lung injury model, where the inflammatory response (TNF-α) and oxidative stress were not affected by gelatin [22]. A recently published investigation has also indicated that NF-κB activation, proinflammatory cytokines levels, ICAM-1 mRNA expression and myeloperoxidase activity were not affected by modified fluid gelatin in a polymicrobial sepsis model [17] – whereas, like other artificial colloids, gelatin impairs firm leukocyte adhesion to the endothelium *in vitro *[23], and applied in the priming fluid gelatin reduces the contact activation of complement cascades by binding to fragment Ba [24]. Nevertheless an increased TNF-α release was demonstrated after the incubation of blood with gelatin *in vitro *[25], but *in vivo *investigation has revealed that gelatin does not alter PMN function [26].

Despite numerous studies having been published concerning the influence of colloids on inflammation, only few comparative studies exist. Reducing the endothelial adhesion of PMNs, dextran was reported to be more potent than HES in leukocyte-related reperfusion injury, and in contrast to HES the anti-inflammatory effect of dextran developed even in nondilutional microdose administration [8].

On the basis of experimental data it may be hypothesized that dextran attenuates the inflammatory response following cardiac surgery. There are, however, no exact clinical data in the literature that would support the anti-inflammatory effect of dextran following cardiac surgery. Our objective was the investigation of the effects of dextran-70 compared with gelatin as a control, on the levels of serum procalcitonin, on the inflammatory cytokine response, the markers of endothelial damage, myocardial ischaemia-reperfusion injury and haemodynamics after CPB. Our hypothesis was that administration of dextran reduces the level of inflammatory mediators and cardiac troponin-I (cTr-I) at the most important timepoints.

## Materials and methods

With permission from the ethical committee of the hospital, 40 patients undergoing elective first-time CPB – 32 patients undergoing coronary artery revascularization (coronary artery bypass grafting (CABG)), eight patients undergoing aortic valve replacement (AVR) – were involved in this prospective, randomized, double-blind study after individual consent was obtained. The setting of the study was single institutional. Two experienced anaesthesiologists and two experienced surgeons were involved in the study. Exclusion criteria were as follows: 'redo' operation, hepatic disease, renal dysfunction, immunologic disease, steroid treatment, intake of aspirin or other cyclooxygenase inhibitor within 7 days prior to surgery, or known allergy to volume expanders used in the study. None of the patients received volatile anaesthetics, steroids or aprotinin and haemofiltration was not used either. No shed mediastinal blood was retransfused during the postoperative period.

Two groups were formed following computerized randomization. Twenty patients (CABG, 17 patients; AVR, three patients) were given dextran-70 (6%; average molecular weight 70,000 Da) infusion (Macrodex; Pharmalink, Inc., Upplands Väsby, Sweden) (group A), while in the control group 20 patients (CABG, 15 patients; AVR, five patients) were given oxypolygelatin (5.5%; average molecular weight 30,000 Da) infusion (Gelifundol; Biotest Pharma, Inc., Dreieich, Germany) (group B). Following the induction of anaesthesia, artificial colloid was administered using infusion pumps (Model 591; IVAC, Inc., San Diego, CA, USA). After the application of hapten inhibition by 20 ml dextran-1 (Promit; Fresenius Kabi, Inc., Norge AS, Norway), dextran-70 infusion was used at the dose of 7.5 ml/kg for 30 minutes before CPB, and at a dose of 12.5 ml/kg for 14 hours following the cessation of CPB. Gelatin was infused by the same body-weight-based volume as dextran. The indication of the colloid administration was volume substitution. Depending on the actual haemodynamic and volume status, crystalloid infusion was administered together with a fixed dose of colloid.

Anaesthesia was carried out by a standardized total intravenous method. Premedication was achieved with midazolam. For the induction of anaesthesia, midazolam, propofol in a target controlled infusion perfusion device using Diprifusor™ (Alaris Medical Systems, Hampshire, UK), alfentanil and pipecuronium were used, while propofol (target controlled infusion) and alfentanil were given to maintain anaesthesia. Anticoagulation was maintained with heparin (initial value 300 IU/kg) to keep the activated clotting time longer than 400 seconds. Protamine was administered in a 1:1 ratio based on the initial heparin bolus (necessary to achieve the target activated clotting time). Cardiopulmonary bypass was carried out in normothermia, with the use of a roller pump (Cobe Cardiovascular, Inc., Arvada, Colorado USA), with pulsatile flow rate of 2.4 l/min/m^2 ^and a membrane oxygenator (Affinity™ NT 541; Medtronic, Inc., Minneapolis, MN, USA). Antegrade, cold, crystalloid cardioplegia (modified Bretschneider solution) injected into the aortic root was used for myocardial protection. The Pulsion PiCCO™ (Pulsion Medical Systems, Inc., München, Germany) device was used for haemodynamic monitoring.

Packed red blood cell administration was applied when the haemoglobin level was less then 90 g/l, or during CPB when haemoglobin was below 70 g/l. Postoperative complications were defined as follows: cardiovascular complication (low cardiac output with cardiac index <2.2 l/min/m^2 ^after volume infusion, requiring the use of positive inotrop agents and/or intraaortic balloon pump); perioperative myocardial infarction with typical electrocardiogram changes and creatine kinase MB >75 U/ml (three times the upper limit of the reference range); acute lung injury (prolonged ventilation, PaO_2_/FiO_2 _ratio <200); acute renal failure (serum creatinine >230 μmol/l); neurologic complications (stroke, ischaemic insults); gastrointestinal complications (ischaemia, bleeding); and infections.

Arterial blood samples were taken from the indwelling femoral artery cannula at the following time intervals: t1, before anaesthesia; t2, 10 minutes after CPB; t3, 2 hours after CPB; t4, 4 hours after CPB; t5, 24 hours after CPB; and t6, 44 hours after CPB. At the same timepoints, the following haemodynamic parameters were registered: heart rate, arterial blood pressure, cardiac index, stroke volume index, stroke volume variation, systemic vascular resistance index (SVRI), intrathoracic blood volume index, and extravascular lung water index. The haematocrit (packed cell volume), haemoglobin and blood cell count were measured at all time intervals. Determination of the plasma concentration of inflammatory mediators – interleukins (IL-6, IL-6r, IL-8, IL-10) and soluble adhesion molecules (soluble endothelial leukocyte adhesion molecule-1 (sELAM-1), soluble ICAM-1) – was carried out by ELISA (DIACLONE Research™, Besançon, France) complying with the technologic regulations of the manufacturer. Blood samples were centrifuged with a cooled centrifuge at 1,000 × *g *for 15 minutes. Centrifuged plasma was stored at -86°C.

Three plasma samples were analysed for cytokine and soluble adhesion molecule levels with the sampling timepoints based on the kinetics of the single mediators according to the data in the literature. In each case the first measurement point was the preoperative control value, the second was the expected maximal value of the given mediator after cardiac surgery, while the third measurement point was the value corresponding to the dropoff phase [1,7]. The t1, t3 and t4 samples were analysed for IL-6 and IL-6r, while for IL-8 and IL-10 determinations the t1, t2, and t3 samples were used. Monitoring of the adhesion molecules was done as follows: sELAM-1 at the t1, t4 and t5 timepoints, and soluble ICAM-1 as the t1, t5 and t6 samples. The average of the duplicated ELISA test results was corrected using the following equation: corrected concentration = noncorrected concentration × actual plasma fraction/plasma fraction of the t1 sample. Procalcitonin was measured using an immunoluminometric assay (reference range 0–0.5 ng/ml; LUMItest™; BRAHMS GmbH, Berlin, Germany). For the measurement of serum C-reactive protein the immune turbidimetric method was used (reference range 0–10 mg/l; DIALAB™, Vienna, Austria). cTr-I was measured by a two-site immunoluminometric assay (reference range 0–0.03 ng/ml; LIAISON™ Troponin I; DiaSorin S.p.A, Saluggia, Italy). For procalcitonin measurements we used the t1 and t5 blood samples, while for C-reactive protein the t1, t5 and t6 samples were used, and for cTr-I the t1, t4, t5 and t6 samplings were used.

### Statistical analysis

Statistical analysis was performed by SPSS for Windows 9.0 software (SPSS Inc., Chicago, Illinois, USA). After obtaining the results for 22 patients a midterm analysis was performed to calculate the necessary total sample size – Altman's nomogram [27] was used after calculating the standardized difference of the inflammatory mediators, setting the statistical power at 80% and choosing a 5% significance level. In the case of IL-8, IL-10, ICAM-1 and procalcitonin, the standardized difference and the necessary sample size were (0.924) 36, (0.886) 39, (1.326) 19 and (1.041) 29, respectively. According to the midterm analysis the sample size was determined to be 40 patients.

For the comparison of the basic data, the chi-squared test and Student's *t *test were used. We checked the parameters for normal distribution by the Kolmogorov–Smirnov test. If the result proved significant, the nonparametric test was used consecutively. Among inflammatory markers and cTr-I, nonparametric methods were used (Kruskal–Wallis test, Friedman test); and for haemodynamic parameters the general linear model repeated-measurement analysis (analysis of variance) with Bonferroni adjustment for multiple comparison was used for the analysis of the intraindividual and interindividual differences. Any difference was regarded statistically significant if *p *< 0.05. The results are displayed in the form of the mean ± standard deviation, as the median (range), or as a box-plot where the median, 25–75% and 2.5–97.5% percentile ranges are depicted.

## Results

Past medical and perioperative data of the two groups are presented in Table 1. No operative mortality, low cardiac output, lung injury, renal failure, neurological complications, gastrointestinal complications, surgical intraoperative problems or wound healing problems occurred among the patients involved in the study. Myocardial infarction had developed in three cases, one case in group A and two cases in group B. Positive inotrope drug was not applied after the operation, and no differences were found in the frequency of vasodilator therapy (*p *= 0.584) or β-blocker therapy (*p *= 0.333) between the groups. The total postoperative mediastinal and pleural drainage proved to be higher in group A. Nevertheless, the amount of the transfused red blood cell units did not differ between the two groups. There was also no significant difference between the preoperative and the 44-hour postoperative haematocrit values, which indicates that the red blood cell loss was approximately the same in both groups.

**Table 1:** Anamnestic and perioperative data

	Group A	Group B	*p *value^a^
Age (years)	61.1 ± 6.5	62.5 ± 7.6	0.535
Gender (male/female)	13/7	14/6	0.736
Body mass index (kg/m^2^)	28.7 ± 3.9	28.9 ± 3.8	0.821
Hypertension (%)	47.2	52.8	0.292
Diabetes mellitus (type I/type II/impaired glucose tolerance) (%)	2/4/1	2/3/1	0.733
Preoperative ejection fraction (%)	55.6 ± 12.6	56.1 ± 9.7	0.906
EuroScore (log) (%)	2.5 ± 1.2	2.9 ± 1.4	0.405
Amount of plasma substitute (ml)	1626 ± 212	1606 ± 205	0.725
Amount of crystalloids (ml)	4172 ± 660	4107 ± 665	0.765
Number of anastomoses (coronary artery bypass graft)	3.5 ± 1.0	3.5 ± 0.9	0.991
Aortic Xclamp (min)	53 ± 13.8	59 ± 18.1	0.403
Cardiac surgery on cardiopulmonary bypass duration (min)	83 ± 23.7	90 ± 29.3	0.401
Operation time (min)	249 ± 64.5	258 ± 61.7	0.634
Minimum rectal temperature (°C)	34.8 ± 0.6	35.0 ± 0.5	0.274
Preoperative packed cell volume	0.40 ± 0.034	0.39 ± 0.040	0.723
Postoperative 44 hours packed cell volume	0.30 ± 0.035	0.30 ± 0.035	0.623
Postoperative drainage (44 hours, ml)	818 ± 286	588 ± 179	0.005
Red blood cell transfusion (U)	1.8 ± 1.3	1.6 ± 1.2	0.548
Extubation time (hours)	9.2 ± 4.1	9.3 ± 4.6	0.894
Intensive care unit stay (hours)	54 ± 23	47 ± 6	0.209
Hospital stay (days)	9.9 ± 2.4	8.9 ± 1.3	0.114

Data presented as the mean ± standard deviation. ^a^Chi-squared test or Student's *t *test.

**Figure 1:**
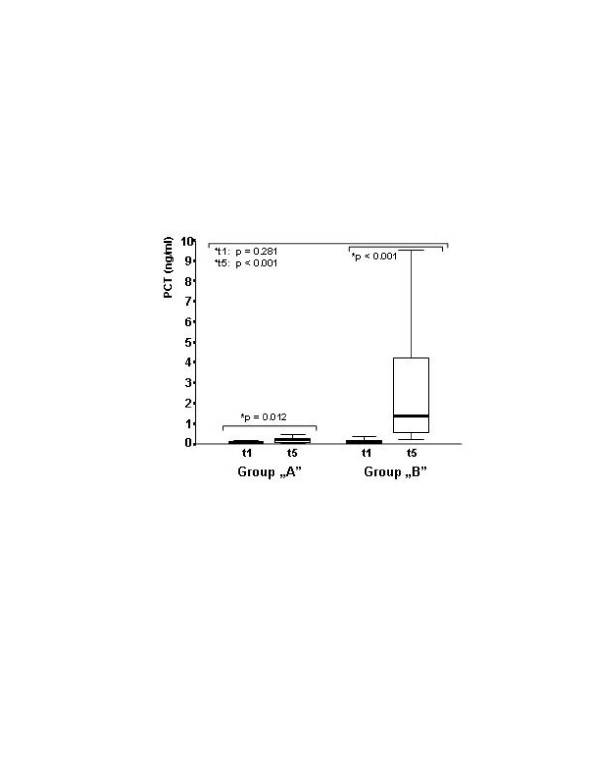
Procalcitonin plasma levels before operation and 24 hours after cardiopulmonary bypass. Procalcitonin (PCT) plasma levels in the treated and control groups, before operation (t1) and 24 hours after cardiopulmonary bypass (t5). Significant elevation was found in both groups (*Friedman tests). After the operation, procalcitonin was lower in group A. The between-group difference was significant (^+ ^Kruskal–Wallis test).

**Figure 2:**
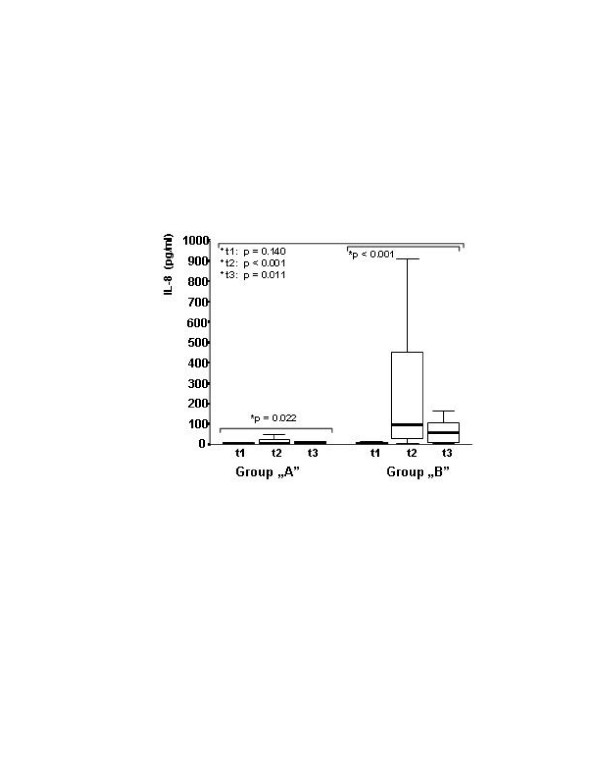
IL-8 plasma levels before operation and after cardiopulmonary bypass. IL-8 plasma levels in the treated and control groups, before operation (t1) and 10 minutes (t2) and 2 hours (t3) after cardiopulmonary bypass. Significant elevation was found in both groups (*Friedman tests). At t2 and t3 the IL-8 plasma levels were lower in group A. The between-group differences were significant (^+^Kruskal–Wallis test).

**Figure 3:**
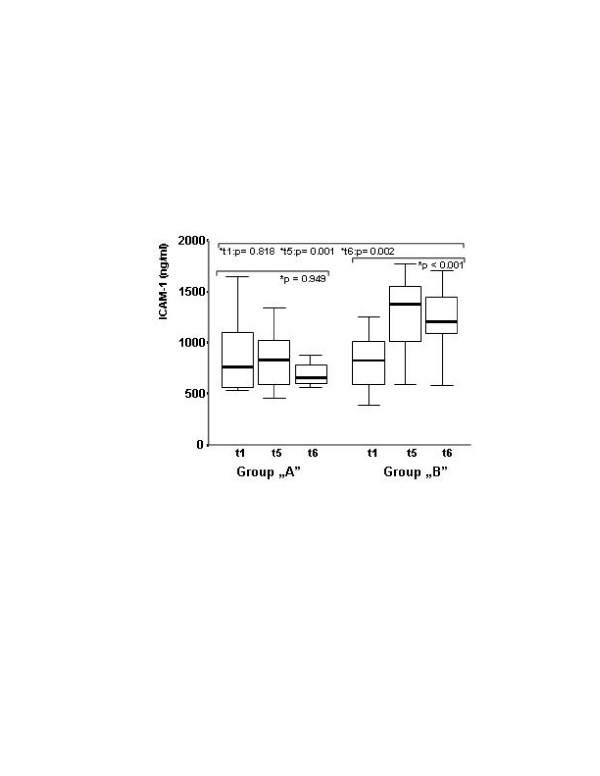
Soluble intercellular adhesion molecule 1 plasma levels before operation and after cardiopulmonary bypass. Soluble intercellular adhesion molecule 1 (ICAM-1) plasma levels in the treated and control groups, before operation (t1) and 24 hours (t5) and 44 hours (t6) after cardiopulmonary bypass. No elevation was found in group A, but the elevation was significant in group B (*Friedman tests). The between-group differences were significant after cardiac surgery on cardiopulmonary bypass (^+^Kruskal–Wallis test).

The peak level of procalcitonin was lower in group A (Figure 1.). The procalcitonin level was below 2 ng/ml for all of the patients in group A, but it increased in 40% of the patients in group B with a peak level higher than 2 ng/ml (*P *= 0.001). IL-8 level elevation was moderate in group A in contrast to the increase experienced in the control group, which is a well-known characteristic of CPB. The between-group difference in IL-8 was significant after CPB (Figure 2). The same kinetics were found for IL-10 (Table 2). The preoperative concentration of sELAM-1 was higher in group A, and did not increase any further, in contrast to the control group that started at a low preoperative value and subsequently rose significantly (Table 2). The level of soluble ICAM-1 did not show any increase in group A. On the contrary, there was a significant and characteristic increase in the control group. The between-group differences for soluble ICAM-1 were significant after CPB (Figure 3). No significant difference was found in C-reactive protein, IL-6 or IL6r between the groups. The cTr-I levels were lower in group A, especially at the t5 timepoint (Table 2).

**Table 2:** Results of the inflammatory mediators and cardiac troponin I

	t1	t2	t3	t4	t5	t6	*p*
C-reactive protein (mg/l)							
Group A	3.7 (1.0–22.6)				79.9 (50.0–131.7)	112.0 (61.1–177.6)	<0.001^†^
Group B	2.6 (0.6–10.5)				87.6 (52.4–143.0)	131.0 (71.0–228.0)	<0.001^†^
	*p *= 0.068*				*p *= 0.092*	*p *= 0.168*	
IL-6 (pg/ml)							
Group A	1.6 (0.4–21.8)		95.0 (13.6–405.1)	46.3 (16.1–149.5)			<0.001^†^
Group B	2.0 (0.4–60.6)		130.5 (21.7–353.8)	49.2 (14.8–214.5)			<0.001^†^
	*p *= 0.107*		*p *= 0.829*	*p *= 0.482*			
IL-6r (ng/ml)							
Group A	43.6 (1.7–125.0)		47.4 (0.7–109.5)	56.2 (25.2–226.3)			0.949^†^
Group B	40.7 (15.6–94.6)		42.4 (22.2–100.5)	50.2 (13.2–104.9)			0.861^†^
	*p *= 0.607*		*p *= 0.914*	*p *= 0.304*			
IL-10 (pg/ml)							
Group A	1.9 (0.2–24.0)	47.2 (2.3–476.6)	7.2 (1.3–90.9)				<0.001^†^
Group B	2.6 (0.8–9.7)	209.7 (16.3–814.3)	56.1 (3.6–225.1)				<0.001^†^
	*p *= 0.136*	*p *= 0.001*	*p *= 0.001*				
Soluble endothelial leukocyte adhesion molecule-1 (ng/ml)							
Group A	88.6 (49.8–194.5)			88.5 (14.4–189.6)	72.7 (8.9–163.1)		0.058^†^
Group B	49.0 (18.3–144.1)			130.7 (33.0–360.7)	72.6 (16.7–224.7)		<0.001^†^
	*p *< 0.001*			*p *= 0.033*	*p *= 0.957*		
Cardiac troponin-I (ng/ml)							
Group A	0.02 (0.01–0.022)				0.22 (0.07–0.85)	0.13 (0.03–0.75)	<0.001^†^
Group B	0.01 (0.01–0.016)				0.66 (0.10–1.28)	0.19 (0.03–0.80)	<0.001^†^
	*p *= 0.520*				*p *= 0.018*	*p *= 0.097*	

Data presented as the median (range). t1, before anaesthesia; t2, 10 minutes after cardiac surgery on cardiopulmonary bypass (CPB); t3, 2 hours after CPB; t4, 4 hours after CPB; t5, 24 hours after CPB; t6, 44 hours after CPB. ^†^Intraindividual differences (Friedman test), *between-group differences (Kruskal–Wallis test).

No difference was found between CABG and AVR patients in the inflammatory markers or cTr-I, except for ICAM-1 in group B, which was higher among AVR patients before operation (*p *= 0.047) and at the 24-hour timepoint (*p *= 0.023).

Patients with postoperative atrial fibrillation or pacemaker rhythm in the VVI mode (group A, five patients; group B, four patients) were excluded from the analysis of haemodynamic data. The cardiac index and stroke volume index were higher while the SVRI figures were lower in group A, with an observed statistical power of 83%. The intrathoracic blood volume index, stroke volume variation, heart rate, arterial blood pressure (systolic) (*p *= 0.787), arterial blood pressure (diastolic) (*p *= 0.771) and extravascular lung water index (*p *= 0.326) did not differ between the two groups (Table 3).

**Table 3:** Results of the haemodynamic data

	t1	t2	t3	t4	t5	t6	*p *value^a^
Heart rate (l/min)							0.925
Group A	60 ± 12	76 ± 14	78 ± 13	84 ± 18	84 ± 7	92 ± 10	
Group B	59 ± 8	72 ± 9	78 ± 12	81 ± 12	87 ± 12	97 ± 12	
Stroke volume index (ml/m^2^)							0.026
Group A	38.5 ± 6.5	33.5 ± 9.1	31.9 ± 8.2	32.8 ± 9.0	36.9 ± 13.8	36.1 ± 7.4	
Group B	36.4 ± 5.7	30.2 ± 5.8	27.6 ± 8.0	27.6 ± 7.5	31.3 ± 10.0	30.1 ± 5.0	
Cardiac index (l/min/m^2^)							0.010
Group A	2.3 ± 0.45	2.4 ± 0.39	2.4 ± 0.63	2.7 ± 0.63	3.0 ± 0.98	3.4 ± 0.94	
Group B	2.2 ± 0.36	2.2 ± 0.31	2.0 ± 0.41	2.2 ± 0.44	2.6 ± 0.38	2.9 ± 0.49	
Stroke volume variation (%)							0.873
Group A	1.5 ± 0.8	2.3 ± 3.3	3.4 ± 5.3	2.2 ± 1.8	4.5 ± 11.5	2.0 ± 0.7	
Group B	2.6 ± 3.9	3.1 ± 2.9	4.9 ± 8.5	2.8 ± 3.1	2.5 ± 1.5	2.5 ± 1.0	
Intrathoracic blood volume index (ml/m^2^)							0.387
Group A	861 ± 132	829 ± 129	875 ± 120	882 ± 122	923 ± 137	975 ± 136	
Group B	845 ± 153	811 ± 151	831 ± 192	831 ± 169	871 ± 128	919 ± 214	
Systemic vascular resistance index (dyn s/cm^5 ^m^2^)							0.005
Group A	2,663 ± 414	2,031 ± 492	2,701 ± 604^†^	2,418 ± 534	2,076 ± 514	2,052 ± 430	
Group B	2,732 ± 424	2,279 ± 428	3,499 ± 964	2,902 ± 659	2,470 ± 505	2,227 ± 507	

Data presented as the mean ± standard deviation. t1, before anaesthesia; t2, 10 minutes after cardiac surgery on cardiopulmonary bypass (CPB); t3, 2 hours after CPB; t4, 4 hours after CPB; t5, 24 hours after CPB; t6, 44 hours after CPB. ^a^Between-group differences, general linear model repeated measurement analysis (analysis of variance). ^†^*p *< 0.05 (with Bonferroni adjustment for multiple comparison).

## Discussion

For the first time according to the literature, we have shown in the present investigation that dextran-70 reduces the inflammatory cytokine response, reduces the peak levels of serum procalcitonin and reduces the peak levels of the markers of endothelial activation or damage during the inflammatory activation following CPB. Earlier investigation had indicated that dextran-70 reduces complement-3 activation product levels during cardiopulmonary bypass [28], but subsequently it was revealed that fresh frozen plasma, which was applied in the control group in large volume, contains a high level of complement-3 [29].

The exact mechanism of the anti-inflammatory effect of dextran is still unclear. Intravital studies on haemodilution in controlled ischaemia have shown that dextran reduces leukocyte adhesion onto the endothelium. The inhibition of leukocyte–endothelium interaction already occurs in the pharmacological microdose of dextran, independently of the haemodilution effect [8]. On the basis of recently published experimental data, dextran inhibits neutrophil adhesion by a neutrophil-dependent mechanism, regulating integrin function rather than interfering with endothelial cell activation [23]. In the same experimental setting, gelatin represented a similar effect on neutrophil adhesion to that of dextran; therefore, it can be supposed that other mechanisms should also be of importance. The termination of a neutrophil-mediated inflammatory response is effected through apoptosis of the neutrophils. When PMNs were exposed to dextran in whole blood samples, significant apoptosis was demonstrated. By these effects, human PMN survival was found to decrease [30]. Inflammatory mediators also modulate PMN survival. IL-8 plays a major role in the markedly reduced rate of programmed cell death of neutrophils after CPB [31]. In the present investigation we have demonstrated the attenuation of IL-8 release by dextran-70. The free radical scavenger effect of dextran is also well known [8]. This action may contribute to the reduction of endothelial activation and injury after CPB, as clearly demonstrated in our investigation.

From the good predictive value of procalcitonin [6] and its blunted kinetics in the dextran-treated group in our study, one can suppose that dextran-70 may influence the patients' outcome after CPB. Further large-scale studies are required to confirm this assumption.

In our investigation a similarly blunted peak level of IL-8 was found after dextran-70 administration as that reported earlier by Wan and colleagues on coronary patients operated on without CPB [32]. IL-8 plays an important role in the formation of the myocardial and pulmonary ischaemia-reperfusion injury developing after the use of CPB [3,7,33]. Its serum concentration correlates with postoperative cTr-I values [33]. The peak plasma IL-8 level also correlates with the magnitude of the proximal tubular injury following CABG surgery [34]. On the basis of our results, a lower IL-10 peak concentration occurred proportionally with the IL-8 peak level when dextran infusion was used, which indicates that no imbalance developed between proinflammatory and anti-inflammatory responses. High doses of aprotinin or a heparin-coated CPB tubing set also reduce IL-10 production following CPB [33,35]. Steroids increase the concentration of IL-10 with the consequences of immune suppression [35,36].

We found no significant action of dextran-70 on the levels of C-reactive protein, IL-6 and IL-6r, but the peak concentration of IL-6 and C-reactive protein was lower and that of IL-6r was higher in the group administered dextran. Previous investigations proved that IL-6 [33,37] and C-reactive protein [2] levels did not differ in CABG and off-pump coronary artery bypass surgery, but reduced IL-6 levels occurred after minimally invasive direct coronary artery bypass surgery, which suggests that operative trauma itself rather than CPB or ischaemia-reperfusion injury initiates the release of these factors [2]. These results suggest that dextran can reduce the inflammation resulted from either the use of CPB or ischaemia-reperfusion injury rather than from operative trauma.

There is an increasing body of evidence that the damaging effect of ischaemia-reperfusion in the heart is related to inflammatory processes [3] and that attenuation of the inflammatory response improves myocardial outcome following cardiac surgery [36]. The pattern of cardiac cTr-I release is similar after CABG and after valve surgery, but off-pump coronary artery bypass surgery results in less elevation than CABG using CPB [38]. Troponin-I release was reduced in the patient group where dextran-70 was applied, which may indicate that dextran-70 decreases the ischaemia-reperfusion injury after cardiac operation. The difference in cTr-I was rather small between the two groups, and therefore its clinical relevance must be confirmed in a large-scale study.

In our investigation dextran infusion has increased the stroke volume index independently of the preload by the reduction of the systemic vascular resistance index. Although the intrathoracic blood volume index and stroke volume variation did not differ between the groups it cannot be excluded that the observed differences in haemodynamics are partly due to the different volume effects of dextran and gelatin – whereas on the basis of our sELAM-1 and soluble ICAM-1 results, dextran infusion reduces the degree of the endothelial damage and activation. Particularly high preoperative sELAM-1 plasma concentration figures were found in the dextran group in contrast to the control group, indicating higher preoperative endothelial dysfunction among these patients. The levels of sELAM-1 and ICAM-1 significantly increased in the control group, but did not change in those patients who received dextran. This effect on the activation and damage of the endothelium may explain dextran's favourable influence on the vasomotor regulatory disturbance. Cardiopulmonary bypass alters vasomotor regulation, reducing the endothelium-dependent relaxation [39]. The nitric oxide production is significantly reduced up to 6 hours after CPB [40], which coincides with the time interval where the main difference in the SVRI was found in our study.

For the statistical analysis, six series of observations were represented over time to show the haemodynamic status at every examined timepoint of the inflammatory mediators. Using Bonferroni correction for multiple comparison, these many timepoints have resulted in a very high correction factor, increasing the possible statistical error, which may mask real differences. Although the observed statistical power was high, particularly in case of the SVRI, the clinical relevance of these finding must be justified in a study investigating a larger population.

The administration of HES 130/0.4 has also been investigated on the inflammatory response in other patient populations (for example, in patients undergoing major abdominal surgery). The IL-6, IL-8 and soluble ICAM-1 release was found to be lower in the HES-treated group, but the concentration of sELAM-1 was similar in the HES-treated and in the lactated Ringer's solution-treated groups [20]. Although HES does possesses anti-inflammatory properties [17,18], as the authors concluded these results were probably due to differences in microcirculation, because crystalloid-based volume therapy may worsen the microcirculatory blood flow and tissue oxygenation despite sufficient haemodynamic conditions [41]. The microhaemodynamic status was not measured in our investigation, but previously gelatin had been shown to increase cerebral blood flow velocity and to improve cutaneous microcirculation, more expressively than HES, during haemodilution (to a haematocrit level of 30.0%). That effect on microcirculation was related inversely to haematocrit, which means that after autologue blood retransfusion the parameters of cutaneous microcirculation have worsened [42]. Similarly to dextran, in our investigation HES was shown to diminish the endothelial activation or damage in critically ill patients. The soluble ICAM-1 and sELAM-1 levels did not increase following HES administration compared with the increase of these molecular levels in the control group treated with human albumin (20%).

On the basis of comparative animal experiments investigating the effects of colloids on leukocyte-endothelial interaction we have investigated the presumably most effective colloid, dextran-70, during CPB and we have proved its anti-inflammatory effect. Having said that, questions still remain of whether dextran has any advantage over other colloids in the anti-inflammatory properties in clinical situations, and whether it results in real clinical benefit on patient outcome. Further clinical studies are required to compare the anti-inflammatory effect of dextran with that of HES, and large-scale clinical studies must justify that the reduction of inflammation by dextran can be translated into clinical benefit. Another question to be raised is whether dextran administrated in a microdose is, or is not, able to reduce the inflammation in clinical situations of systemic inflammatory response syndrome, as in experimental studies [8], because microdose administration eliminates the antihaemostatic effects.

Blood loss was higher in the dextran group than the control group, which can be explained by the higher antihaemostatic potential. This difference, however, is surprisingly not clinically significant, since the number of red blood cell transfusions and haematocrit levels did not differ.

The limitation of this study is that gelatin, or any other colloid, administrated in the control group cannot be considered neutral concerning the inflammatory response. An infusion possessing anti-inflammatory effects is more acceptable for the control group than an infusion with proinflammatory features. Although gelatin administration did not influence neutrophil infiltration, NF-κB activation, proinflammatory cytokines levels, ICAM-1 mRNA expression and myeloperoxidase activity in a septic model [17], a moderate favourable effect on inflammation was indicated, reducing the Ba fragment of the complement cascade [24] and impairing the firm leukocyte adhesion in an *in vitro *model [23]. Certain albumin batches possess well-documented proinflammatory actions (for example, activating E-selectin, ICAM-1, vascular cell adhesion molecule 1), which seriously question the relevance of human albumin as a valid control for studies concerning inflammation [43]. Also, the use of lactated Ringer infusion is associated with significant neutrophil activation [44] and early expression of endothelial E-selectin and P-selectin [45]. Another limitation of this study is the single institutional setting. There were no differences in morbidity, mortality or length of intensive care unit stay between the groups but the low number of patients in our study does not allow one to conclude on the clinical outcome parameters.

## Conclusion

To the best of our knowledge our investigation is the first to show that dextran-70 reduces the inflammatory cytokine response, the liberation of some soluble adhesion molecules and the peak level of procalcitonin following cardiac operations. These results suggest that dextran can reduce the inflammation resulting from either the use of CPB or ischaemia-reperfusion injury rather than from operative trauma. Further large-scale clinical studies are required to demonstrate this effect on patient outcome after cardiac surgery.

## Key messages

• Dextran-70 reduces the inflammatory activation after CPB.

## Abbreviations

AVR = aortic valve replacement; CABG = coronary artery bypass grafting; CPB = cardiac surgery on cardiopulmonary bypass; cTr-I = cardiac troponin I; ELISA = enzyme-linked immunosorbent assay; HES = hydroxyethyl starches; ICAM-1 = intercellular adhesion molecule-1; IL = interleukin; NF = nuclear factor; PMN = polymorphonuclear leukocyte; sELAM-1 = soluble endothelial leukocyte adhesion molecule-1; SVRI = systemic vascular resistance index; TNF = tumour necrosis factor.

## Competing interests

The authors declare that they have no competing interests.

## Authors' contributions

KG conceived the study, participated in the design, coordination, measurements and acquisition of data, performed the statistical analysis, drafted the manuscript and obtained sponsorship. AB made a substantial contribution to the execution of the study and acquisition of data. NA and LB made a substantial contribution to the design of the study, interpretation of the data and provided critical review of the manuscript. NA participated in the coordination of the study. GK participated in the study design and helped to write the manuscript. VG and JG carried out the immunoassays, discussed the results and provided a critical review of the manuscript. TK carried out laboratory measurements, discussed the results and provided a critical review of the manuscript. All authors read and approved the final manuscript.
